# High-Resolution Imaging of the Retinal Nerve Fiber Layer in Normal Eyes Using Adaptive Optics Scanning Laser Ophthalmoscopy

**DOI:** 10.1371/journal.pone.0033158

**Published:** 2012-03-12

**Authors:** Kohei Takayama, Sotaro Ooto, Masanori Hangai, Naoko Arakawa, Susumu Oshima, Naohisa Shibata, Masaaki Hanebuchi, Takashi Inoue, Nagahisa Yoshimura

**Affiliations:** 1 Department of Ophthalmology and Visual Sciences, Kyoto University Graduate School of Medicine, Kyoto, Japan; 2 NIDEK, Gamagori, Japan; 3 Central Research Laboratory, Hamamatsu Photonics K.K., Hamakita, Japan; University Hospital La Paz, Spain

## Abstract

**Purpose:**

To conduct high-resolution imaging of the retinal nerve fiber layer (RNFL) in normal eyes using adaptive optics scanning laser ophthalmoscopy (AO-SLO).

**Methods:**

AO-SLO images were obtained in 20 normal eyes at multiple locations in the posterior polar area and a circular path with a 3–4-mm diameter around the optic disc. For each eye, images focused on the RNFL were recorded and a montage of AO-SLO images was created.

**Results:**

AO-SLO images for all eyes showed many hyperreflective bundles in the RNFL. Hyperreflective bundles above or below the fovea were seen in an arch from the temporal periphery on either side of a horizontal dividing line to the optic disc. The dark lines among the hyperreflective bundles were narrower around the optic disc compared with those in the temporal raphe. The hyperreflective bundles corresponded with the direction of the striations on SLO red-free images. The resolution and contrast of the bundles were much higher in AO-SLO images than in red-free fundus photography or SLO red-free images. The mean hyperreflective bundle width around the optic disc had a double-humped shape; the bundles at the temporal and nasal sides of the optic disc were narrower than those above and below the optic disc (*P*<0.001). RNFL thickness obtained by optical coherence tomography correlated with the hyperreflective bundle widths on AO-SLO (*P*<0.001)

**Conclusions:**

AO-SLO revealed hyperreflective bundles and dark lines in the RNFL, believed to be retinal nerve fiber bundles and Müller cell septa. The widths of the nerve fiber bundles appear to be proportional to the RNFL thickness at equivalent distances from the optic disc.

## Introduction

The sensory retina is composed of nine contiguous layers, linked to each other by synaptic connections. Evaluation of the retinal nerve fiber layer (RNFL) is crucial for detecting and managing glaucoma, which is a progressive optic nerve disease characterized by a loss of the RNFL. Although red-free fundus photography is the standard approach for examining the RNFL, changes in the RNFL are often not detectable until there is more than 50% nerve fiber loss [1]. Another limitation is the difficulty in obtaining fundus photographs with sufficient quality for interpretation, especially in eyes with a hypopigmented fundus or myopia, when background reflection is high and contrast is low. With the advent of optical coherence tomography (OCT), cross-sectional imaging of the RNFL has improved detection of damage to the RNFL. Relatively high diagnostic sensitivity and specificity for glaucoma detection has been demonstrated using time-domain OCT (TD-OCT) and spectral-domain OCT (SD-OCT) RFNL thickness measurements [2], [3]. Studies using commercially available OCT have not, however, provided sufficiently clear images of individual nerve fiber bundles to identify a specific structural abnormality that underlies the pathogenesis of glaucoma.

OCT and other imaging modalities such as scanning laser ophthalmoscopy (SLO) fail to provide sufficiently detailed images of RNFL microstructure primarily because of aberrations in ocular optics. These aberrations can be compensated for by using imaging systems that incorporate adaptive optics (AO), consisting of wavefront sensor that measures aberrations in ocular optics and a deformable mirror or a spatial light modulator to compensate for these aberrations in living eyes [4]. Adding AO to imaging systems such as flood-illuminated ophthalmoscopes, SLO equipment, or OCT has allowed researchers to identify individual cone photoreceptors, presumed nerve fibers, and blood cells [5]–[20]. However, to date, the spatial distribution of the nerve fiber layer has not been investigated using AO equipment.

In the present study, we used an AO-SLO system developed by the authors to conduct high-resolution imaging of the RNFL of the posterior pole and around the optic disc in normal eyes by constructing a large-scale map of RNFL images.

## Methods

All investigations adhered to the tenets of the Declaration of Helsinki, and the study was approved by the institutional review board and the ethics committee of Kyoto University Graduate School of Medicine. The nature of the study and its possible consequences were explained to study candidates, after which written informed consent was obtained from all participants.

### Participants

Self-reported ophthalmologically healthy subjects of ≥20 years of age were recruited for the study. Ocular examination at the first visit included autorefractometry/keratometry, uncorrected and best-corrected visual acuity measurements using a 5-m Landolt chart, axial length measurements using an IOL Master (Carl Zeiss Meditec, Dublin, CA), slit-lamp examinations, intraocular pressure measurements using a Goldmann applanation tonometer, dilated fundoscopy, stereo fundus photography, red-free SLO imaging, and visual field testing using the Humphrey 24-2 Swedish Interactive Thresholding Algorithm (HFA; Carl Zeiss Meditec).

Exclusion criteria were as follows: 1) contraindication to dilation; 2) Snellen equivalent best-corrected visual acuity worse than 20/20; 3) spherical equivalent refractive error of >5.0 or <−6.0 diopters; cylindrical refractive error of <−3.0; 4) intraocular pressure ≥22 mm Hg; 5) unreliable HFA results (fixation loss ≥20%, false positives, or false negatives ≥20%); 6) abnormal HFA findings suggesting glaucoma according to the Anderson and Patella criteria [21]; 7) any abnormal visual field loss consistent with ocular disease; 8) a history of intraocular surgery; 9) evidence of vitreoretinal diseases; and 10) evidence of optic nerve or RNFL abnormality, diabetes mellitus, or other systemic diseases that might affect the eye.

Patients were first examined by an ophthalmologist using dilated fundoscopy to rule out retinal or optic nerve diseases. In addition, two ophthalmologists examined all of the acquired stereo color fundus photographs and reached a consensus on whether each eye had evidence of glaucomatous optic neuropathy or other optic nerve abnormalities and on whether each eye had evidence of retinal disease.

### Adaptive Optics Scanning Laser Ophthalmoscopy System

**Figure 1:**
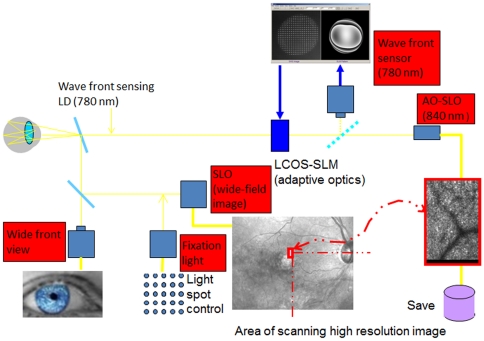
Optical system diagram. The adaptive optics scanning laser ophthalmoscopy (AO-SLO) system comprises 4 primary optical subsystems: the AO subsystem including the wavefront sensor, the high-resolution confocal SLO imaging subsystem, the wide-field imaging subsystem, and the pupil observation subsystem for initial alignment of the subject's pupil with the optical axis of the AO-SLO system by adjusting the chin rest. The AO subsystem incorporates a liquid-crystal spatial light modulator (LC-SLM) based on liquid crystal-on-silicon (LCOS) technology. The LC-SLM and wavefront sensor are controlled using a custom software to reduce the wavefront errors. The results of high-resolution imaging are linked to the results of the wide-field imaging subsystem, which are obtained by the line-scan SLO system.

The utility of incorporating a wide-field SLO with an AO-SLO was reported by Burns et al. [22], [23]; we have designed and constructed our AO-SLO system based on the same scheme with certain simplifications [16]–[18]. The AO-SLO system comprises 4 primary optical subsystems, the AO subsystem including the wavefront sensor, the high-resolution confocal SLO imaging subsystem, the wide-field imaging subsystem, and the pupil observation subsystem for initial alignment of the subject's pupil with the optical axis of the AO-SLO system by adjusting the chin rest (Fig. 1).

The AO subsystem contains a liquid-crystal-on-silicon spatial light modulator (LCOS-SLM), a Shack-Hartmann wavefront sensor, and software. The light source for wavefront sensing is a 780-nm laser diode (LD: the light power is 70 µW at the subject's pupil). Custom software controls the liquid-crystal spatial-light modulator and the wavefront sensor in order to reduce the residual wavefront aberrations arising from the AO-SLO system and the subject's eye. The LCOS-SLM consists of a parallel-aligned liquid crystal layer, a multilayer dielectric mirror, and active-matrix circuits with pixelated electrodes [24]. The number of pixels is 792×600, and the pixel size is 20 µm×20 µm. The multilayer dielectric mirror was designed to have 99% reflectivity in the wavelength range of the LD laser and the SLO. While the stroke of the LCOS-SLM is almost 1 wavelength, the effective phase stroke of more than 20 wavelengths can be achieved using the phase-wrapping technique [25]. The wavefront sensor consists of a lens array and a high-speed camera [26]. The lens array has 25×25 square lenslets in a 10 mm×10 mm active sensor area. The software performs closed-loop AO control at a rate of 10 Hz. Aberration sensing and correction was performed within a circular area. The diameter of the area at the corneal plane was approximately 5.5 mm, and the number of lenslets in the area was approximately 225.

The SLO subsystem uses an 840-nm superluminescent diode (SLD) with 50-nm full width and the illuminating source at half-maximum (the light power at the subject's pupil is 210 µW). The custom computer software reads the output of an avalanche photodiode detector in synchronization with both the horizontal raster scans made by a resonant scanner (SC-30, Electro-Optical Products Co., NY) and the vertical scans created by a galvano scanner (6230H, Cambridge Technology, MA) in order to achieve an image acquisition rate of 50 frames per second (each image is 512×320 pixels and covers an area of 3.0°×1.9° in width and height, respectively) by using both the forward and return sweeps of the resonant scanner [27].

This subsystem is optically designed to cancel intrinsic aberrations. The defocusing aspect of the aberrations of the whole eye is manually corrected with a Badal optics unit, which is mounted on the translation stage; other aberrations are compensated for by the AO system, which allows for the achievement of the diffraction-limited projection of the fiber tip of the light source onto the arbitrary layer in the retina. Although LCOS-SLM of the AO subsystem works only for 1 specific wavelength in principle, we experimentally confirmed significant improvements in lateral resolution and image contrast.

The principle of line-scan SLO was employed as a wide-field imaging subsystem, in which we use a 910 nm SLD as a light source and a one-dimensional charge-coupled device as a detector and a confocal slit, which suppresses scattering of the reflection from the retina. The image acquisition rate is 50 frames per second and the angular field of view is 28° and 24° along the horizontal and vertical directions, across which the retinal region can be shifted arbitrarily.

The AO-SLO system is confocal, allowing us to create high-contrast “en face” images, in any plane in the living retina.

### Measurement of Widths of the Hyperreflective Bundles

**Figure 2:**
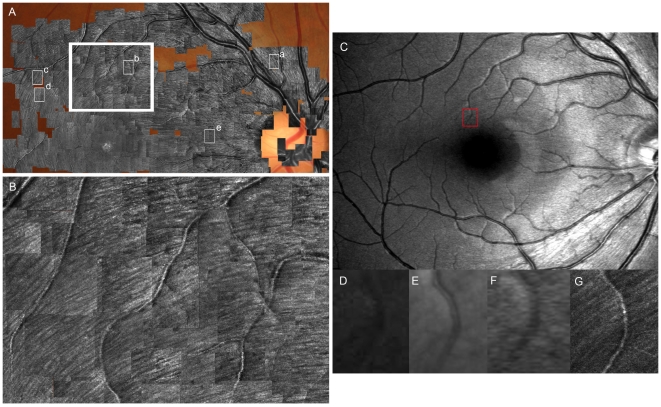
Images of the normal retinal nerve fiber layer (RNFL) using AO-SLO compared with red-free fundus photography and red-free SLO. **A**, Wide-field montage of high-resolution AO-SLO images (1.5°×1.5°) within a 30° arc from the foveal center. AO-SLO images show many hyperreflective bundles in the RNFL. The hyperreflective bundles above and below the fovea appear in an arched shape from the temporal periphery on either side of a horizontal dividing line to the optic disc. The hyperreflective bundles on the nasal side of the fovea arch from the fovea to the optic disc. Small white boxes (**a**–**e**) indicate the area of high-magnification AO-SLO images in Fig. 5. **B**, Magnified view of the area outlined in large white box in **A** showing individual hyperreflective bundles. **C**, Red-free SLO image of the same eye obtained with an F-10 (NIDEK, Gamagori, Japan). The hyperreflective bundles on AO-SLO (**A**, **B**) correspond with the direction of the striations on the SLO red-free image. **D**, Magnified blue-channel fundus photography image of the area inside the box in **C**. **E**, Magnified red-free SLO image (HRA2; Heidelberg Engineering, Heidelberg, Germany) of the area inside the box in **C**. **F**, Magnified red-free SLO image (F-10) of the area inside the box in **C**. **G**, Magnified AO-SLO image of the area inside the box in **C**. The resolution and contrast of the bundles are much higher in the AO-SLO images (**G**) than in red-free fundus photography (**D**) or the red-free SLO images (**E**, **F**).

For each eye, AO-SLO images were obtained at multiple locations in the posterior polar area and a circular path with a diameter of 3–4 mm around the optic disc. All participant eyes were dilated for examination, and AO-SLO imaging was performed by focusing on the surface of the RNFL. Then, offline, a montage of AO-SLO images was created by selecting the area of interest and generating each image to be included in the montage from a single frame, without averaging. The degree of correspondence of each montage to the area of interest was verified by comparing the AO-SLO image with the wide-field images for that eye. To create a large-scale montage of AO-SLO images (Fig. 2A and Fig. S1), the total acquisition time was 65 minutes (Fig. 2; by using a 1.5°×1.5° field of view) or 5 minutes (Fig. S1; by using a 3.0°×1.9° field of view), and an automated image-stitching algorithm was applied.

**Figure 3:**
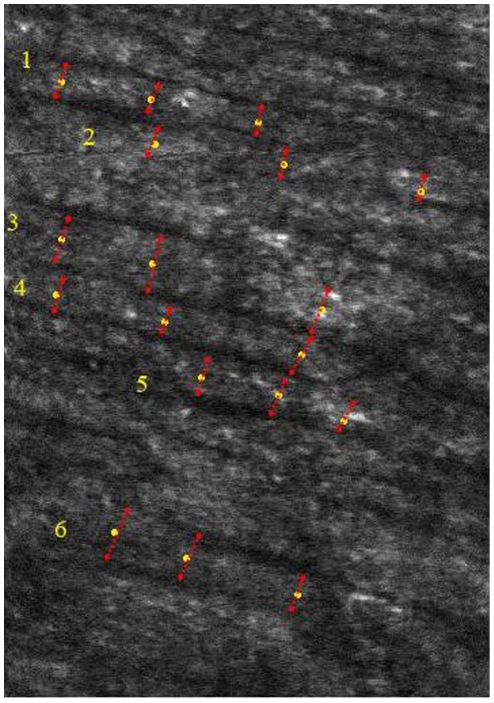
Measurement of the width of hyper-reflective bundles. To measure the width of individual hyper-reflective bundles, 3–8 bundles were randomly chosen from 1 AO-SLO image (1.5°×1.5°). The digital caliper tool was used to measure the width at a minimum of 3 points in 1 bundle by 2 independent experienced graders (i.e., the bundle width of this area was defined as the mean width of 18 points).

To measure the width of individual hyper-reflective bundles, 3–8 bundles were chosen from 1 AO-SLO image (Fig. 3). The digital caliper tool built into Image J (National Institutes of Health, Bethesda, MD) was used to measure the width at a minimum of 3 points in 1 bundle (i.e., the bundle width of each area was defined as the mean width of 9–24 points) by 2 independent experienced graders (SO and NA) who were blinded to the bundle location and other clinical information regarding the eyes. In each area of each eye, 11.5±2.7 points were measured and averaged. To obtain accurate scan lengths, we corrected for the magnification effect in each eye using the adjusted axial length method devised by Bennett et al. [28]. The width of each hyperreflective bundle was determined as the mean width acquired from these images. If the values were significantly different between the graders, the opinion of the first author (KT) was invited and the results were discussed until consensus was reached.

### Comparison of SLO red free images and AO-SLO images

Six experienced ophthalmologists who were masked to the image information performed independent expert comparisons of pairs of SLO red free images and AO-SLO images obtained from the same area of the retina. Three areas were selected randomly from 1 participant, and 60 images of each type were compared. The scoring results were as follows [29]: for detecting hyper-reflective bundles, AO-SLO image had better contrast evidently: AO-SLO scored 4 and red free scored 0. AO-SLO had slightly better image: AO-SLO scored 3 and red free scored 1. The two images had same contrast: AO-SLO scored 2 and red free scored 2. Red free images had slightly better image: AO-SLO scored 1 and red free scored 3. Red free images had better contrast evidently: AO-SLO scored 0 and red free scored 4.

### Comparison of the number of peaks per plot profile

**Figure 4:**
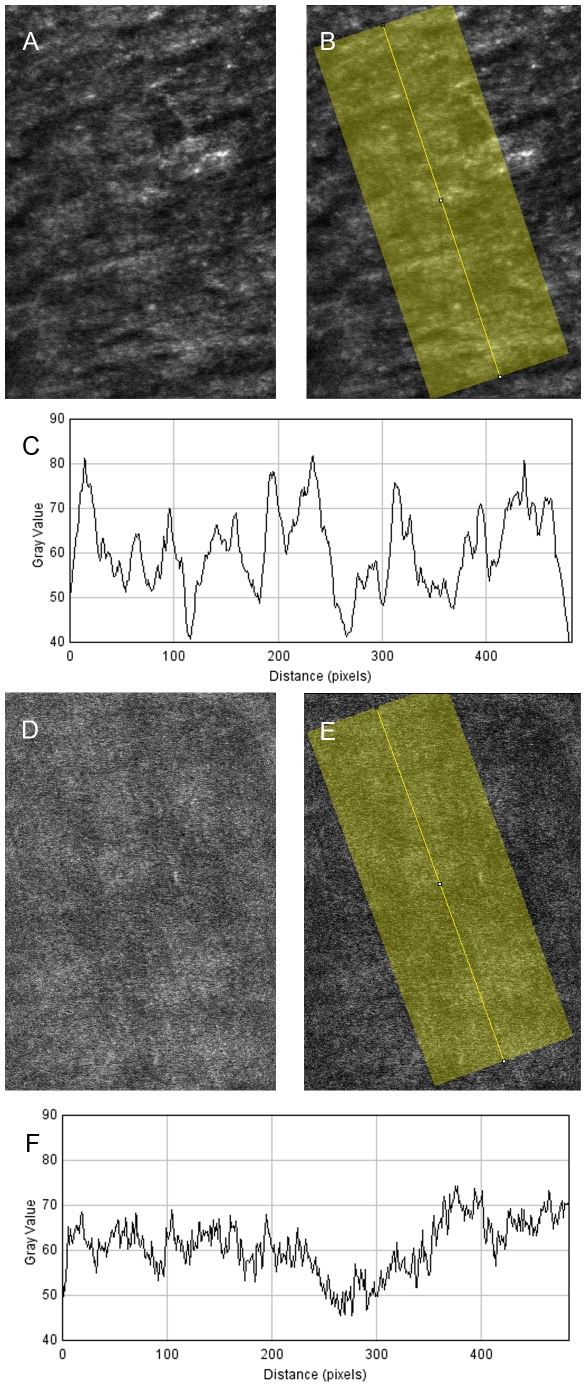
Comparison of the number of peaks of plot profile between AO-on images and AO-off images. **A**, **B**. AO-on image focused on the RNFL. Mean gray value of images was plotted (190 pixel width) along a line that vertically crosses the hyper-reflective bundles. **C**. Plot profile of gray value along the yellow line of **B**. The number of peaks, which was defined as more than 20 gray values compared to the neighboring baseline, was 8. **D**, **E**. AO-off image of same area. **F**. Plot profile of gray value along the yellow line of **E**. The number of peaks was zero.

To evaluate the effect of AO on image quality (for detecting hyperreflective bundles), the number of peaks per plot of AO-on and AO-off SLO images were compared. AO-off SLO images were acquired without using an adaptive optics system; images were taken with remaining aberrations in ocular optics. AO-on SLO images were acquired using an adaptive optics system that compensated for the aberration in the ocular optics. The number of peaks per plot profile between AO-on and AO-off images was compared using the plot profile tool built into Image J (National Institutes of Health). One area was selected randomly from each participant, and a total of 20 images from each participant were compared. Mean gray value of images was plotted (190 pixel width) along a line that vertically crosses the hyper-reflective bundles (Fig. 4), and the number of peaks was counted. A peak was defined as more than 20 gray values compared to the neighboring baseline.

### SD-OCT: RNFL thickness measurement

SD-OCT examinations were performed on all eyes using the Spectralis HRA+OCT (Heidelberg Engineering, Dossenheim, Germany). First, horizontal and vertical line scans through the fovea center were obtained at a 30° angle, followed by serial horizontal scans with an examination field size 30×25°and a 3.4 mm circle scan from the optic disc. The RNFL thickness was manually measured with the digital calipers built into the SD-OCT system.

The Spectralis HRA+OCT has built-in software that calculates circumpapillary RNFL thickness, and we used this function to measure the thickness of the RNFL on circle scan images in each of the areas within a 3.4 mm circle from the optic disc. The lines delineating the sectors were automatically drawn by the software.

### Statistical Analyses

For comparing bundle width variables among areas, Kruskal-Wallis test was used. For inter-observer measurements, intraclass correlation coefficients (ICC) were obtained. The image quality score and the number of peaks per plot profile were compared using the Wilcoxon signed-rank test. Statistical analyses were performed using the SPSS17 statistics software program (SPSS Inc., Chicago, IL). A *P* value<0.05 was considered statistically significant.

## Results

Twenty normal eyes of 20 Japanese subjects (12 men, 8 women) were included in the study. The ages of the enrolled subjects ranged from 22 to 54 years (average, 36.8 years). The axial length ranged from 22.96 to 27.06 mm (average, 25.01 mm).

**Figure 5:**
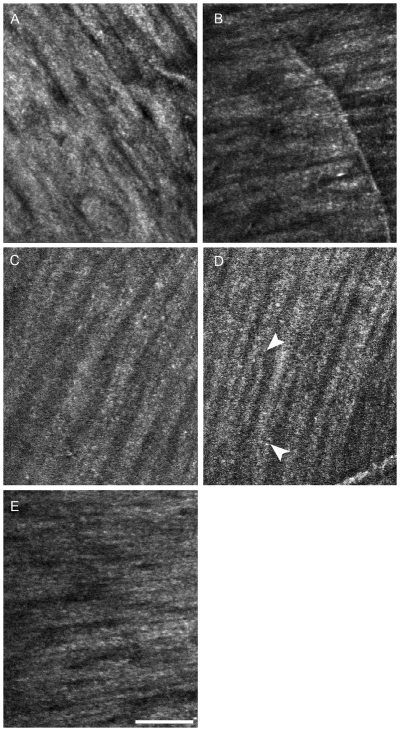
High-magnification RNFL images using AO-SLO of the area indicated by white boxes in Fig. 2 (**a**–**e** in Fig. 2 corresponds to **A**–**E** in Fig. 5). **A**, Image near the optic disc indicated by a in Fig. 2. **B**, Image 2 mm above the fovea. **C**, **D** Images at the temporal raphe. Bridges can be seen among the hyperreflective bundles (*arrows*). **E**, Image of the area around the papillomacular bundle. The dark lines among the hyperreflective bundles are narrower around the optic disc (**A**) than at the temporal raphe (**C**, **D**). The hyperreflective bundles on the nasal side of the fovea (**E**) are thinner than those above (**B**) or below the fovea. Scale bar = 100 µm (A–E).

In all of the eyes, the AO-SLO images showed many hyperreflective bundles in the RNFL (Fig. 2A,B). The hyperreflective bundles above and below the fovea appeared from the temporal periphery on either side of a horizontal dividing line to the optic disc in an arched shape. The hyperreflective bundles nasal to the fovea arched from the fovea to the optic disc. The dark lines among the hyperreflective bundles were narrower around the optic disc than in the temporal raphe (Fig. 5A,C,D). The hyperreflective bundles nasal to the fovea were narrower than those above or below the fovea (Fig. 5B,E). Some of the hyperreflective bundles had bridges among the bundles (Fig. 5D). The hyperreflective bundles corresponded with the direction of the striations on SLO red-free images in all eyes (Fig. 2A,C). However, the resolution and contrast of the bundles were much higher in the AO-SLO images than in red-free fundus photography or red-free SLO images (Fig. 2D–G). In expert comparisons of image quality, the score for AO-SLO images was 3.95±0.22 and the score for SLO red free images was 0.05±0.22 (*P*<0.001, Wilcoxon signed-rank sum test). To evaluate the effect of AO on image quality (for detecting hyperreflective bundles), the number of peaks per plot profiles of AO-on and AO-off SLO images were compared. The numbers of peaks per plot profile was 6.75±2.63 for images with AO and 0.96±1.77 for images without AO (*P*<0.001, Wilcoxon signed-rank sum test). (Fig. 4)

The reproducibility of the hyperreflective bundle width measurements was evaluated through an inter-observer ICC; the ICC was 0.950 for measurement of bundle width.

**Figure 6:**
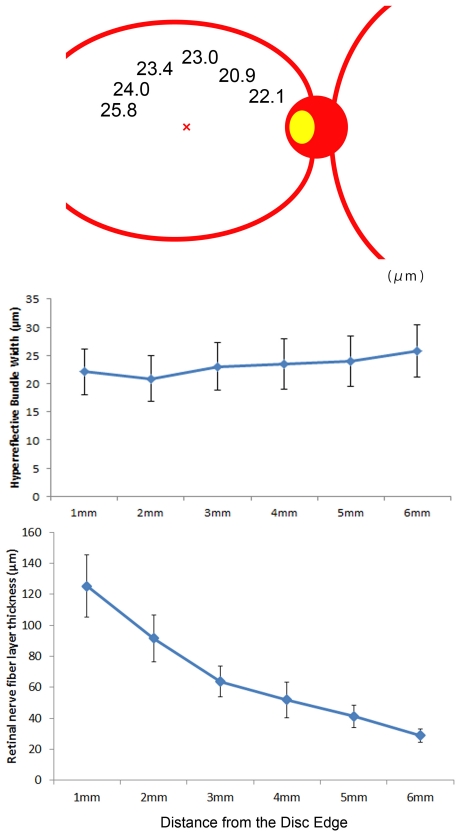
Mean widths of the hyperreflective bundles at various distances from the optic disc. (Top,Middle) Mean widths of the hyperreflective bundles in normal eyes at 1∼6 mm from the optic disc. Measurement points are 1 mm, 2 mm, 3 mm, 4 mm, 5 mm, and 6 mm from the optic disc. There are no significant differences among the bundle widths at these distances. (Bottom) RNFL thickness was measured by SD-OCT in normal eyes at the corresponding area on AO-SLO measurement points. Note that the RNFL thickness on SD-OCT deceases in proportion as distances from the optic disc increase, whereas the hyperrefrective bundle width remains constant. The error bars are SDs of 20 normal eyes.

The mean widths of the hyperreflective bundles at various distances from the optic disc are shown in Fig. 6. There were no significant differences among these bundle widths (*P* = 0.100, Kruskal-Wallis test). Though the RNFL thickness on SD-OCT deceased in proportion as distances from the optic disc increased, the hyperrefrective bundle width remained constant (Fig. 6).

**Figure 7:**
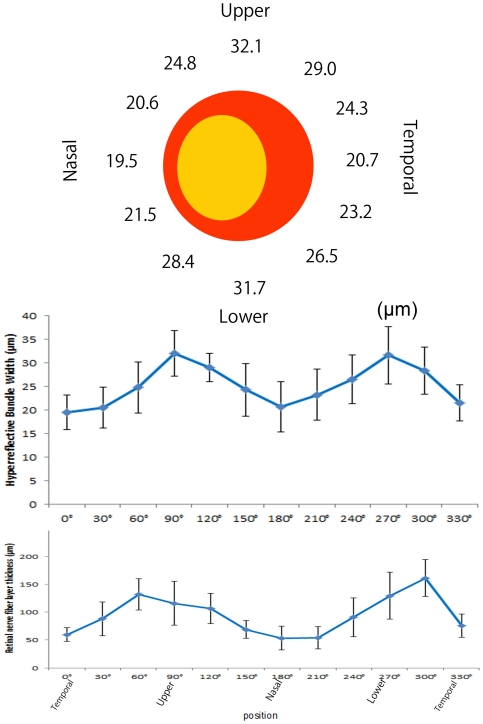
Mean widths of the hyperreflective bundles at the same distance around the optic disc. (Top, Middle) Mean widths of the hyperreflective bundles in normal eyes at a diameter of 3.4 mm centered on the optic disc. The hyperreflective bundles on the temporal and nasal sides of the optic disc are narrower than those above and below the optic disc; thus, the bundle widths around the optic disc have a double-humped shape. (Bottom) RNFL thickness measured by SD-OCT in normal eyes at a diameter of 3.4 mm centered on the optic disc. Note that the RNFL thickness measured by SD-OCT around the optic disc also has a double-humped shape. The error bars are SDs of 20 normal eyes.

The mean widths of the hyperreflective bundles at a diameter of 3.4 mm centered on the optic disc are shown in Fig. 7. The hyperreflective bundles on the temporal and nasal sides of the optic disc were narrower than those above and below the optic disc (*P*<0.001, Kruskal-Wallis test). The bundle width around the optic disc had a double-humped shape (Fig. 7). The RNFL thickness measured by SD-OCT around the optic disc also had a double-humped shape (Fig. 7), and the RNFL thickness correlated with the hyperreflective bundle widths on AO-SLO (*P*<0.001, rs = 0.556, Spearman's rank correlation coefficient).

## Discussion

Because morphological changes in the optic disc and the RNFL often precede observable visual field loss in glaucoma [30], early diagnosis of glaucoma requires an intimate knowledge of the configuration of the optic disc and the distribution of the RNFL in normal subjects. Many previous studies have analyzed RNFL thickness in normal subjects using various imaging techniques, such as confocal SLO (Heidelberg Retina Tomography [HRT]; Heidelberg Engineering, Germany) [31], scanning laser polarimetry (GDx Nerve Fiber Analyzer; Laser Diagnostic Technologies, Inc, San Diego, CA), [31]–[33] and OCT [2], [3], [31], [32], [34]–[37]. In these reports, RNFL thickness was measured at the optic disc using HRT, at a diameter of 3.2 mm or a diameter of 1.5–1.75 times that of the optic disc using GDx, at a diameter of 3.40 or 3.46 mm using TD-OCT, or at a diameter of 2.2–4.0 mm using SD-OCT. However, variations in the width of retinal nerve fiber bundles with distance and direction from the optic disc had not been evaluated.

Morphological features of nerve fiber bundles have been identified using various imaging techniques such as fundus photography, SLO, and OCT [1], [38]–[43]. Quigley et al. compared histological features of the RNFL and clinical fundus photography [1], and suggested that the striations seen in fundus photography are derived from various reflections from nerve fiber bundles and the glial septa. Kawaguchi et al. reported that the radial striations in SLO under argon blue laser illumination are the reflex from the bundle of retinal ganglion cell axons [40]. Using adaptive optics OCT (AO-OCT), Cense et al. [19] and Zawadzki et al. [42] confirmed that the striations seen in C-scan images focused on the RNFL were retinal nerve fiber bundles. In the present study using AO-SLO, the hyperreflective bundles seen in the inner retina corresponded with the direction of striations seen in red-free SLO images in all eyes, and were consistent with the previously reported histological and AO-OCT features. Thus, it appears reasonable to suggest that the hyperreflective bundles visible on AO-SLO images represent individual retinal nerve fiber bundles. The present study, in addition to confirming the presence of retinal nerve fiber bundle features such as fiber bridges among the bundles and the direction of striations, determined the widths of normal retinal nerve fiber bundles in the posterior pole and around the optic disc.

The resolution and contrast of the bundles were much higher in AO-SLO images than in red-free fundus photography or red-free SLO images. Using OCT, the thickness of the nerve fiber layer can be measured; however, commercially available OCT or red-free SLO do not provide sufficiently clear images of individual nerve fiber bundles to identify any specific structural abnormality that may underlie the pathogenesis of glaucoma. The advantage of the AO-SLO system is that it enables us to detect and analyze individual nerve fiber bundles, which may be applied to early-stage glaucoma detection or to monitor disease progression.

AO-SLO revealed dark lines among the hyperreflective bundles. It has been established that the axons of ganglion cells are enclosed by tissue tunnels composed of elongated processes of Müller cells that transmit, rather than reflect, light through the retina [44]. Thus, the dark lines among the hyperreflective bundles on AO-SLO appear to represent Müller cell septa. These dark lines were narrower around the optic disc than in the temporal raphe, consistent with histological findings that Müller cell septa are wider in the temporal raphe [45], [46].

The morphological characteristics of retinal nerve fiber bundles have been studied histologically [45]–[52]. Ogden reported that in the primate retina, the arcuate nerve fiber bundles near the disc contain proportionately more fibers than those in the periphery [45]. In another study, Ogden showed that the RNFL near the disc was thicker than that far from the optic disc [46], confirmed by SD-OCT [37]. In contrast, the present study found that the width of hyperreflective bundles did not differ significantly at various distances from the optic disc (Fig. 6). Thus, retinal nerve fiber bundles nearer the optic disc may be thicker, whereas the width of retinal nerve fiber bundles may be constant. These findings suggest that convergence of ganglion cell axons from the retinal periphery toward the optic disc gives rise to an increasing thickness of the retinal nerve fiber bundles without increasing their width.

In the present study, the widths of the hyperreflective bundles around the optic disc had a double-humped shape (Fig. 7); hyperreflective bundles at the temporal and nasal side of the optic disc were narrower than those above and below the optic disc. Various studies using confocal SLO, scanning laser polarimetry, and OCT have determined that the thickness of the circumpapillary RNFL (cpRNFL) has a double-humped shape in normal eyes and that the cpRNFL is thicker above and below compared to the temporal and nasal sides of the optic disc [34]–[37], which was also seen in the current study. In addition, the RNFL thickness measured by SD-OCT correlated with the hyper-reflective bundle widths on AO-SLO. These findings suggest that the width of the retinal nerve fiber bundles may be proportional to the RNFL thickness at the same distance from the optic disc.

Our study has several limitations: (1) We tried the automated segmentation of nerve fiber bundles on AO-SLO images, but found it difficult. Thus, in the current study, in each area of each eye, 11.5±2.7 points were measured by 2 independent, experienced graders and the value was averaged. The inter-observer reproducibility of the hyperreflective bundle width measurements was high (the ICC was 0.950). In addition, our results were comparable to the histological findings of the primate retina reported by Ogden et al. (20–40 µm) [45] and Pocock et al. (20.8 µm) [52]. (2) Although the lateral resolution of AO-SLO is superior to that of commercially available SLO or SD-OCT equipment, currently available AO imaging equipment cannot clearly show individual nerve fibers or faint nerve fiber bundles in the temporal raphe. (3) Another limitation of the current study is that the images taken near the optic disc may show stacks of bundles rather than individual bundles; the thickness of the RNFL near the optic disc is considerably larger, and several bundles may lie on top of each other. The depth resolution of the AO-SLO system is 50–100 µm, and as a result, several bundles stacked on top of each other may contribute to the image. However, there were no significant differences in width between bundles near the optic disc or bundles at each distance from the optic disc. In addition, a continuous bundle could be traced from optic disc on a montage of AO-SLO images. Thus, by focusing on the surface of the RNFL, hyper-reflective bundles on AO-SLO seem to represent individual retinal nerve fiber bundles even near the optic disc.

In conclusion, our study shows that AO-SLO can detect retinal nerve fiber bundles over a wide area in normal eyes, and we anticipate using this technology to learn more about RNFL damage associated with changes in visual function caused by diseases such as glaucoma.

## Supporting Information

Figure S1 **Wide-field montage of high-resolution AO-SLO images (3.0°×1.9°) within a 30° arc from the foveal center.** The total acquisition time was 5 minutes in this field of view, and an automated image-stitching algorithm was applied to create the montage.
(TIF)
