# Supplementary figures and images for: High-Resolution Imaging of the Retinal Nerve Fiber Layer in Normal Eyes Using Adaptive Optics Scanning Laser Ophthalmoscopy
Source: PLoS One. 2012 Mar 12;7(3):e33158. doi: 10.1371/journal.pone.0033158 (PMC3299751; doi:10.1371/journal.pone.0033158)

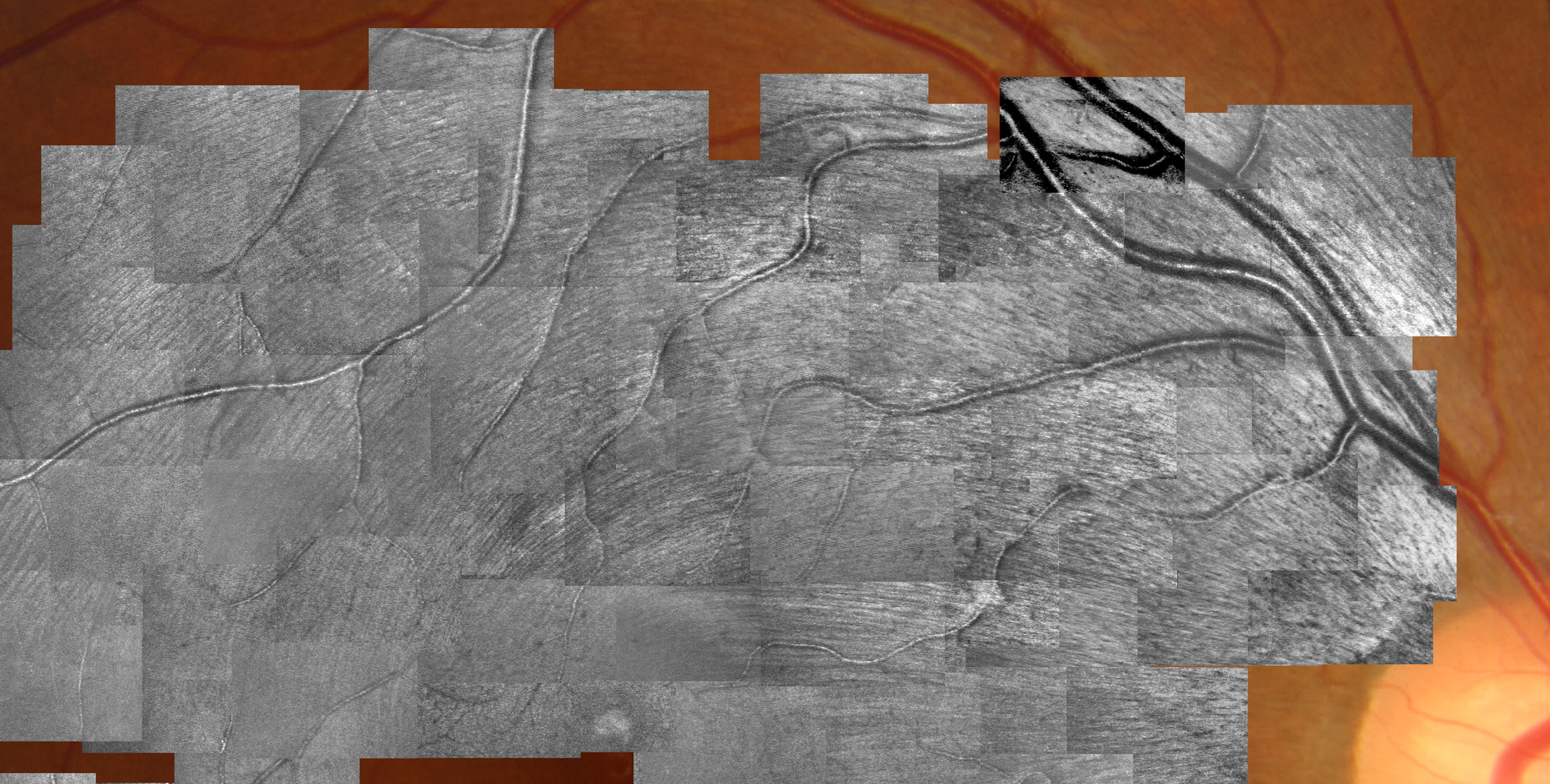

Supplement: Figure S1 — Wide-field montage of high-resolution AO-SLO images (3.0°×1.9°) within a 30° arc from the foveal center. The total acquisition time was 5 minutes in this field of view, and an automated image-stitching algorithm was applied to create the montage. (TIF) [file pone.0033158.s001.tif]
